# New author guidelines and launch of *Short Reports*

**DOI:** 10.1002/prp2.591

**Published:** 2020-04-29

**Authors:** Andrew J. Lawrence, Michael F. Jarvis

**Affiliations:** ^1^ Florey Institute of Neuroscience & Mental Health Melbourne Brain Centre University of Melbourne Melbourne Australia; ^2^ Global Medical Affairs, Abbvie, Inc. North Chicago Illinois

*Pharmacology Research & Perspectives* (*PR&P*) has experienced remarkable growth over the last several years. As a Gold Open Access collaborative journal between the American Society for Pharmacology and Experimental Therapeutics (ASPET), the British Pharmacology Society (BPS), and Wiley & Sons, *PR&P* provides a unique venue for the advancement of all aspects of pharmacological research. In 2019 alone, the journal had a 40% increase in total submissions and 125% increase in direct submissions. Additionally, online downloads for articles published in *PR&P* have increased 10‐fold since 2014. These metrics highlight the scientific interest and impact *PR&P* has generated in the global pharmacological research community.

In order to continue to build upon this success and to further enhance the impact of the journal for its readers and authors we announce the implementation of new **Author Guidelines**. The new *PR&P* **Author Guidelines** are aligned with recently revised author guidelines for the other ASPET and BPS journals^1^, ^2^, ^3^ and support the author‐elected manuscript referral and transfer process at *PR&P*. Importantly, the new **Author Guidelines** will be translated into Japanese, Korean, Mandarin, Portuguese, and Spanish to better assist scientists globally.

The new **Author Guidelines** for *PR&P* have been developed to provide greater clarity and practical guidance for authors to communicate their science with enhanced transparency and rigor. Other new features include the implementation of an Authorship Responsibility form during the submission process, the Resource Identification Initiative (RII) for all key reagents and Data Sharing policy. The authorship responsibility form provides an important mechanism to ensure that all authors agree with and take full responsibility for all aspects of their published work. Both RII and data sharing policies are designed to foster research reproducibility and allow readers to better understand reagent and experimental design choices and analyses.

The new guidelines also provide proscriptive instructions regarding the inclusion of methodological details, statistical analyses, and graphical data presentations. Authors should appreciate that there has been much debate regarding experimental design, statistical analysis and the reporting of research outcomes in recent years.^1^, ^2^, ^3^, ^4^, ^5^, ^6^, ^7^, ^8^ There are several informative commentaries on these aspects of pharmacological research which we encourage authors to carefully read.^1^, ^3^, ^5^, ^6^ The new PR&P Author Guidelines are not designed to serve as a “once size fits all” approach. Rather, the new Author Guidelines provide a framework for authors to present their research in a clear, transparent, and scientifically rigorous fashion.

In addition to the Author Guidelines, we announce here a new feature in *PR&P* called ***Short Reports.*** This new section of *PR&P* is designed to expedite the publishing original research papers that communicate exciting new research findings in all areas of pharmacology. Manuscripts considered for **Short Reports** must be no longer than 2,000 words, present a maximum of 2‐3 figures and/or tables inclusive, and have no more than 12 references. Only manuscripts submitted directly to *PR&P* will be considered for **Short Reports** and the publication charges for these articles will be discounted by 50%. The PR&P Editorial Board is committed to providing authors of **Short Reports** manuscripts with timely and constructive feedback. Authors can expect editorial first decisions within 7 days of submission. Revision invitations will be limited to minor corrections only. **Short Reports** are intended to provide authors a venue to communicate key datasets that form part of a larger research project or represent an important milestone in a topical area of pharmacological research.

We hope that readers and prospective authors will find these new features of *PR&P* to be of value.
